# Reproductive Longevity: Innovative Approaches Beyond Hormone Replacement Therapy

**DOI:** 10.3390/medicina62010157

**Published:** 2026-01-13

**Authors:** Nida Jugulytė, Daiva Bartkevičienė

**Affiliations:** 1 Faculty of Medicine, Vilnius University, LT-03101 Vilnius, Lithuania; 2 Clinic of Obstetrics and Gynaecology, Faculty of Medicine, Institute of Clinical Medicine, Vilnius University, LT-03101 Vilnius, Lithuania

**Keywords:** menopause, fertility preservation, reproductive longevity, ovarian aging

## Abstract

With increasing life expectancy driven by rapid biomedical science advancement, reproductive longevity has become a key concept in women’s health. Preventing reproductive senescence is important not only to extend fertility potential but also to preserve endocrine health, enhance quality of life, and promote healthy aging. The end of ovarian function and fertility is symbolized by menopause, as the most eminent index of reproductive aging. Hormone replacement therapy (HRT) remains the mainstay for managing menopausal symptoms. However, as the use of HRT is often limited, there is a need for safe and effective alternatives. This narrative review summarizes current and emerging approaches targeting different stages of reproductive aging. Both hormonal and non-hormonal therapies for vasomotor and genitourinary symptoms are discussed alongside developing fertility preservation techniques, including oocyte vitrification, ovarian tissue cryopreservation, in vitro follicle maturation, and artificial ovary engineering. Furthermore, evolving and experimental ovarian regenerative strategies, such as stem cell transplantation, intraovarian platelet-rich plasma (PRP) injections, antioxidants, metabolic modulators, telomerase activators, and stem cell-derived extracellular vesicles, offer new prospects for delaying or reversing ovarian aging. Overall, personalized regenerative strategies and innovative solutions may reshape the future of women’s reproductive health and longevity.

## 1. Introduction

At a time when biological aging is increasingly viewed as a modifiable process, reproductive longevity stands at the forefront of women’s health. A biological hallmark of reproductive aging is menopause, characterized by a major physiological transition, including decline in ovarian function, cessation of menstruation, and significant hormonal changes, among which a drop in circulating estrogen is the most prominent [1,2,3]. Even though it is a normal life change, menopause for the majority of women is accompanied by bothersome symptoms, such as hot flashes, vaginal dryness, joint and muscular discomfort, mood changes, sleep disorders, and long-term consequences like osteoporosis and cardiovascular diseases [4,5]. For many women, these physical and mental symptoms impair well-being and quality of life [6,7]. As life expectancy increases, women spend approximately one-third of their lives in the post-reproductive stage, raising the need for comprehensive strategies that extend beyond traditional hormone replacement therapy (HRT). Since the 1960s, when the history of HRT began, it has experienced many ups and downs [8,9]. Even though HRT is widely recommended treatment for menopausal symptoms, its use is often limited by contraindications [10], concerns about breast cancer [11,12] and thromboembolic risks [13], and low patient acceptance [9,14]. As a result, it is essential for healthcare professionals to be aware of full spectrum of other therapeutic options that can serve as either alternatives or complements to HRT [15].

Concurrently, delaying reproductive planning into later in life [16,17], as well as medical conditions such as primary ovarian insufficiency (POI) and anti-cancer therapy, have increased the demand for fertility-preserving strategies [18]. Besides traditional assisted reproductive technologies, novel methods and emerging biotechnologies are redefining possibilities of reproductive medicine [19].

In parallel with these developments, there is a growing search for ways to modulate ovarian aging. Novel pharmacological therapies, such as telomerase activators, rapamycin, antioxidants, and metformin, as well as regenerative approaches, ranging from stem cell transplantation to intraovarian platelet-rich plasma (PRP) injections, aim to offset ovarian aging and prolong reproductive lifespan [20,21,22].

This narrative review attempts to present a summary of current and developing alternatives to HRT, with an emphasis on three key aspects: (1) menopausal symptom management, (2) fertility preservation strategies, and (3) experimental methods to delay ovarian aging.

## 2. Alternative Approaches to Menopausal Symptom Relief

While HRT is an effective therapy for menopausal symptoms, such as genitourinary syndrome of menopause (GSM) and vasomotor symptoms [23,24], for some women it is not an optional treatment due to contraindications, side effects, or personal preference. Therefore, alternatives to HRT have gained a notable prominence in relieving menopausal symptoms. The summary of alternative approaches to menopausal symptom relief is provided in Table 1.

### 2.1. Local Agents

#### 2.1.1. Vaginal Moisturizers and Lubricants

Vaginal moisturizers and lubricants remain the first-line treatment for managing GSM [24,25], with vaginal dryness and dyspareunia being the most prevalent symptoms [26]. Vaginal moisturizers are bioadhesive products that maintain hydration and mimic natural secretion by gradually releasing moisture into the vaginal mucosa. Regardless of sexual activity, they are used regularly, typically two to three times per week [25,27]. Lubricants are topical, fast-acting agents available in water-, silicone-, or oil-based formulations. Unlike moisturizers, they act on the surface to reduce friction and temporarily relieve discomfort during intercourse [24,28].

#### 2.1.2. Hyaluronic Acid

Hyaluronic acid is a natural glycosaminoglycan that preserves tissue hydration, promotes cell proliferation and differentiation, and regulates the inflammatory response [29]. Widely used in topical hydrating gels and intravaginal injections, hyaluronic acid increases moisture and may alleviate symptoms of GSM, such as vaginal dryness, itching, dyspareunia, and dysuria [25]. Clinical trials demonstrate hyaluronic acid as an effective and generally well-tolerated method to improve atrophic symptoms [30,31,32,33,34] and show comparable efficacy to vaginal estrogen as both treatments provide relief of vaginal symptoms, decrease pH, and improve epithelial integrity [31,32,34].

#### 2.1.3. Local Estrogen Therapy

Local vaginal estrogen therapy has been the preferred method for treating postmenopausal women when only vulvovaginal symptoms are present, especially if they are mild [35]. Vaginal estrogen therapy is available in multiple formulations (tablets, rings, capsules, pessaries, creams, gels and ovules) and molecular forms (estradiol, estriol, promestriene, conjugated equine estrogens and estrone) [36]. Low-dose and ultralow-dose estrogens are favored over systemic hormone therapy due to their minimal systemic absorption [37] and efficacy in alleviating symptoms of GSM and restoring vaginal epithelial thickness, elasticity, and pH balance [38]. A 2016 Cochrane review confirmed that vaginal estrogen therapy is effective, with no significant difference among various preparations [39]. Importantly, low-dose vaginal estrogen therapy is generally considered safe, as large randomized trials and observational studies have shown no significant increase in the risk of endometrial hyperplasia, endometrial cancer, venous thromboembolism, cardiovascular disease, or breast cancer [24].

#### 2.1.4. Local Dehydroepiandrosterone Therapy

Dehydroepiandrosterone (DHEA) is a steroid pre-hormone, which becomes the main precursor of androgens and estrogens after menopause [40,41]. Prasterone, a synthetic form of endogenous DHEA, seems to be effective in reducing vaginal dryness and itching, improving vaginal cellular structure, secretions, and epithelial integrity, as well as lowering vaginal pH. Improvement in sexual function has also been observed, including increased libido, decreased dyspareunia, and enhanced desire, arousal, lubrication, orgasm, and satisfaction [35,41,42]. Local DHEA administration, with its effects limited to the vagina, keeps serum sex hormone levels within the postmenopausal range, thus preventing the possibility of endometrial stimulation [43].

#### 2.1.5. Vaginal Oxytocin

Oxytocin, a hormone synthesized in the hypothalamus and released by the posterior pituitary, is well-known for its role in labor induction and lactation. Recent studies have further expanded the scope of its clinical potential beyond obstetrics. Intravaginally administered oxytocin stimulates mucosal blood flow, epithelial cell proliferation, and tissue repair by directly acting on its receptor in the lower genitourinary tract [44]. Randomized controlled trials demonstrated that intravaginal oxytocin gel (400 IU) significantly improved the vaginal maturation index, decreased vaginal pH, alleviated subjective symptoms of GSM [45], and enhanced sexual function [46]. A meta-analysis involving 631 postmenopausal women showed that intravaginal oxytocin significantly reduced clinically assessed vaginal atrophy; however, other outcomes such as vaginal pH, histological atrophy, and dyspareunia did not show significant improvement. Given the limited number and heterogeneity of available studies, further well-designed clinical trials are needed to confirm the effectiveness of intravaginal oxytocin for GSM [44].

### 2.2. Pharmacological Therapy

#### 2.2.1. Ospemifene

Ospemifene, a selective estrogen receptor modulator (SERM), has been approved as an effective non-hormonal therapy option for moderate to severe vaginal dryness and dyspareunia due to GSM [24,25,35,47]. Ospemifene provides an estrogenic effect to vaginal tissues by which the vaginal maturation index improves, pH decreases, epithelial thickness increases, and lubrication and sexual function are enhanced [48,49,50]. Ospemifene is generally well tolerated, with vasomotor symptoms being the most common side effect [25]. Long-term safety data do not indicate increased risk of venous thromboembolism, cardiovascular events, and endometrial or breast cancer [35,51,52].

#### 2.2.2. Selective Serotonin Reuptake Inhibitors and Serotonin/Norepinephrine Reuptake Inhibitors

Vasomotor symptoms, including hot flashes and night sweats, affect up to 90% of peri- and postmenopausal women, often severely impairing their quality of life [53]. Selective serotonin reuptake inhibitors (SSRIs) and serotonin/norepinephrine reuptake inhibitors (SNRIs) seem to be effective in reducing the frequency and severity of hot flashes within the first week of treatment [54,55,56]. Among the SSRIs, paroxetine is considered an effective and well-tolerated first-line drug for vasomotor symptoms, reducing hot flashes by up to 64%. Venlafaxine, a commonly used SNRI, also provides rapid relief, but in some patients may cause adverse effects, such as nausea, dry mouth, constipation and sleep changes [56].

### 2.3. Devices and Regenerative Procedures

#### 2.3.1. Laser Therapy

Recently, fractional microablative CO_2_ and non-ablative erbium:YAG lasers have been extensively studied as regenerative therapies for GSM [57]. The principal effect of both laser types is neocollagenesis, elastogenesis and neoangiogenesis, promoting cell repair, tissue restructuring and rejuvenation, and muscle tone restoration [57,58]. Studies demonstrate that this minimally invasive treatment effectively alleviates both genital and urinary symptoms of GSM and improves sexual function [58,59,60]. Vaginal laser seems to improve Visual Analog Scale, Female Sexual Function Index, and Vaginal Health Index scores [60]. Moreover, evidence suggests that laser therapy can be a safe option for managing GSM among breast cancer survivors [61,62]. To date, most data come from small, short-term studies. Although initial results are promising, long-term data on efficacy and safety are still needed [57].

#### 2.3.2. Platelet-Rich Plasma Vaginal Injections

Platelet-rich plasma is an autologous plasma solution enriched with platelets, typically 4 to 7 times above baseline concentration, and packed with bioactive growth factors and cytokines. Initially used in sports medicine, orthopedics and aesthetic medicine, PRP has recently been explored in gynecology as a novel therapy for GSM due to its regenerative potential [63,64]. By promoting angiogenesis, cell proliferation and differentiation, modulating inflammation, and stimulating tissue repair, PRP may improve vaginal atrophy, lubrication, and sexual function, making the vagina look more youthful [30,63,64,65]. However, future randomized trials are necessary to conclusively prove PRP’s effectiveness in managing GSM [64].

### 2.4. Non-Pharmacological Products

#### Phytoestrogens

Phytoestrogens, a group of plant-derived substances divided into three major subgroups (isoflavones, lignans and coumestans), possess weak estrogenic activity and are popular among women seeking natural alternatives to ease menopausal symptoms [66]. Studies show that phytoestrogens may reduce the frequency of hot flashes [66,67], as well as postmenopausal depressive symptoms [68,69]. However, evidence regarding the effectiveness of phytoestrogens is heterogeneous and inconclusive, with some trials showing small and slow effects or no significant benefit at all [66,67,70].

## 3. Fertility Preservation Strategies

The growing use of medical treatments that impair ovarian function, together with the tendency to postpone motherhood later in life, has led to a rising demand for fertility preservation [71]. Fertility preservation is primarily indicated for women undergoing gonadotoxic treatment, such as chemotherapy or radiotherapy, for hematologic malignancies (e.g., leukemia, lymphoma), breast cancer, sarcoma, and some pelvic cancers [18,72,73,74]. In addition to treatment of malignant diseases, other illnesses, including many autoimmune and hematologic conditions, as well as ovarian diseases, carry the risk of POI [71]. Fertility preservation is also relevant for women wishing to delay childbearing due to personal or socio-economic circumstances, with age being the main threat to their fertility [18,71]. Novel technologies and innovative approaches redefine the possibilities for preserving and extending reproductive potential. Table 2 summarizes the established and emerging fertility preservation strategies discussed in this section.

### 3.1. Oocyte Vitrification

Oocyte cryopreservation, based on vitrification technique, is now a well-established and accessible method for women wishing to postpone motherhood for either personal or medical reasons [74]. The process of oocyte vitrification involves retrieving mature oocytes after ovarian stimulation and rapidly freezing them using ultra-rapid cooling to prevent ice crystal formation [75,76]. Current evidence indicates that vitrification is the most effective method for oocyte cryopreservation, yielding greater survival rates compared to slow freezing [77]. Oocyte vitrification has great success rates when performed before the age of 35, with research showing that vitrifying 15 mature oocytes can result in a live birth probability as high as 85% [78]. Compared to ovarian tissue transplantation, oocyte vitrification is less invasive and carries a minimal risk of the transmission of malignant cells, making it especially appealing for oncology patients [74,79]. Even though studies demonstrate that women undergoing oocyte vitrification for oncological indications have lower implantation and live birth rates compared to those preserving fertility for non-oncological reasons, the difference appears to be attributable to older age rather than malignancy itself [80,81]. Moreover, vitrification is the best option for elective fertility preservation and enables reproductive autonomy by allowing women to delay embryo creation until a suitable partner or donor sperm is available [71]. It is necessary to note that outcomes, including survival rate of oocytes, pregnancy, and cumulative live birth rates, depend on a woman’s age and the number of oocytes; therefore, women should be encouraged to freeze their eggs earlier in life, ideally before the beginning of age-related decline in oocyte quality [71,74].

### 3.2. Ovarian Tissue Cryopreservation

Ovarian tissue cryopreservation is another fertility preservation technique, available to patients for over 20 years and no longer considered experimental [82]. Ovarian tissue cryopreservation is especially suitable for prepubertal girls and women who must begin gonadotoxic treatment immediately, as ovarian stimulation and oocyte retrieval take time [71,79,83]. This technique involves laparoscopic or open surgical removal of ovarian cortical tissue, which is then dissected into small fragments containing primordial follicles and cryopreserved using either slow freezing or vitrification [79]. Upon transplantation—usually orthotopically in the pelvic cavity into the remaining ovary, the ovarian fossa, or near the infundibulopelvic ligament [18]—ovarian activity is restored in more than 95% of cases [71,84], usually within 2 to 8 months, with function lasting up to 7 years [79]. Following ovarian tissue cryopreservation and orthotopic autotransplantation, over 189 neonates have been born worldwide [85], with pregnancy rates ranging from 29 to 41% [86,87,88] and live birth rates between 23 and 57% [86,87,88,89,90]. Heterotopic ovarian graft transplantation, typically performed in the arm, is also possible, especially if done to restore hormone function rather than fertility [18,82]. Moreover, it may even support native ovarian recovery, potentially resulting in live births [82]; however, its effectiveness in achieving pregnancy appears significantly lower than orthotopic transplantation (3% vs. 23%), though data remain limited [91].

Besides fertility preservation, ovarian tissue cryopreservation is experimentally used in restoring endocrine function in peri- and postmenopausal women. Transplanted ovarian tissue is able to respond to the hypothalamic–pituitary–ovarian axis, potentially alleviating menopausal symptoms and improving cardiovascular, bone, and mental health [82]. Studies show that menstruations return in 72–88% of patients after transplantation [89,92]. Importantly, to ensure reserve for future endocrine function, it is best to harvest ovarian tissue by the age of 25 [82].

### 3.3. In Vitro Follicle Maturation

In vitro follicle maturation is an evolving approach whereby primordial follicles retrieved from ovarian tissue are matured in laboratory settings. In vitro follicle maturation is designed to avoid the risk of reintroducing cancer cells, particularly in patients with hematologic malignancies or cancers with high ovarian metastasis potential [82,93]. This technique involves culturing ovarian cortex fragments or separated follicles in dynamic multistep systems that replicate the natural follicular environment [71,82]. Live births have already been reported, and, to date, no increase in genetic abnormalities or cancer has been observed in offspring conceived through in vitro follicle maturation [82].

### 3.4. Artificial Ovary

Artificial ovary, a scaffold containing follicles, is a promising development for fertility preservation, especially in patients at high risk of ovarian metastasis, where ovarian tissue cryopreservation presents a risk of transmitting malignant cells [71,94,95]. Artificial ovary technology involves isolating primordial follicles from fresh or thawed ovarian tissue and embedding them in a biocompatible matrix to recreate the ovary’s three-dimensional structure, as well as a functional ovarian environment [94,96]. This way, follicles can be transplanted without the ovarian stroma, significantly lowering the risk of cancer recurrence [72]. Nevertheless, selecting an optimal 3D matrix for an artificial ovary is challenging, as the scaffold must be biocompatible, immunotolerant, thermally adaptable, and mechanically supportive to ensure follicle survival, growth, and neovascularization [94,95]. Antral follicle growth and successful pregnancies have been observed in animal studies and human trials [71,95,97]. Beyond fertility preservation, artificial ovaries provide a promising alternative to hormone replacement therapy for maintaining physiological steroid hormone production in menopausal women or patients with ovarian dysfunction [95,96]. However, several challenges remain, for example, optimizing follicular survival, improving matrix design, and preventing ischemic post-transplantation damage [94,95].

### 3.5. Artificial Intelligence and Robotics

The field of reproductive medicine has rapidly evolved because of technological advances, such as artificial intelligence and robotics. Artificial intelligence facilitates the analysis of large, complex datasets to improve clinical decision-making and develop personalized treatment protocols [98,99]. The use of artificial intelligence is highly incorporated in assisted reproduction, as it helps select and assess the sperm cells, oocytes and embryos; analyze DNA; personalize ovarian stimulation protocols; and optimize treatment by evaluating age, fertility history, and genetic characteristics [100,101]. Robotics, on the other hand, allows for precise and minimally invasive procedures, improving various reproductive techniques like sperm retrieval, embryo handling, and surgical interventions [98]. Robotic-assisted surgery in ovarian tissue retrieval and transplantation offers enhanced dexterity, improved visualization, and reduced surgical time [102].

## 4. Ovarian Aging as a Driver of Systemic Aging

As a woman ages, ovarian function declines due to progressive loss of oocyte quantity and quality [103,104]. This continuous physiological phenomenon is called ovarian aging. Although strongly age-related, ovarian aging may also be precipitated by pathological conditions leading to diminished ovarian reserve or POI [20].

Ovarian aging is increasingly recognized as a key contributor to systemic aging in women, extending far beyond the loss of fertility [105,106]. The ovary is a central endocrine organ that regulates multiple physiological systems through steroid hormone production. Declining estrogen levels during ovarian aging contribute to increased oxidative stress, inflammation, vascular dysfunction, impaired bone remodeling, and cognitive decline, largely due to the widespread distribution of estrogen receptors throughout the female body. Beyond estrogen depletion, nonestrogenic mechanisms, including senescence-associated release of proinflammatory mediators and low-grade inflammation promotion, further link ovarian aging to systemic pathophysiology [107]. Taken together, these mechanisms help explain why menopausal transition coincides with an acceleration of biological aging and a higher risk of age-related non-communicable diseases, including cardiovascular disease, osteoporosis, neurodegenerative disorders such as Alzheimer’s and Parkinson’s diseases, and metabolic disorders [105,106,107].

At the molecular level, ovarian aging is driven by interconnected hallmarks of aging, including genomic instability, telomere attrition, epigenetic alterations, impaired autophagy, mitochondrial dysfunction, oxidative stress, stem cell exhaustion, nutritional dysregulation, cellular senescence, and chronic inflammation [103,106,108]. These processes not only compromise oocyte quality and accelerate follicular depletion but also overlap with mechanisms underlying chronic age-related diseases. Therefore, reproductive aging is positioned as an integral component of systemic aging, highlighting ovarian aging as a potential therapeutic target for mitigating both reproductive decline and broader age-related morbidity [106].

## 5. Strategies to Delay Ovarian Aging: Experimental and Future Directions

As the understanding of ovarian aging advances, interest in novel strategies aimed at delaying its onset or progression grows as well. Experimental therapies attempt to preserve follicular reserve, stimulate ovarian rejuvenation, improve mitochondrial function, reduce oxidative stress, or support telomere maintenance [104,108]. Table 3 provides an overview of experimental approaches aimed at delaying ovarian aging.

### 5.1. Stem Cell Therapy

Stem cells are undifferentiated cells with the unique ability to self-renew and develop into various specialized cell types, if needed [109]. Therefore, stem cell transplantation can potentially rejuvenate ovaries and treat infertility-related disorders, particularly POI [20,109,110,111]. Mesenchymal stem cells, a type of multipotent adult stem cell, are increasingly recognized in both research and clinical use due to their immunomodulatory properties, ability to differentiate into multiple cell lineages, and lack of ethical constraints compared to embryonic stem cells [110]. Mesenchymal stem cells can be obtained from bone marrow, adipose tissue, endometrium, umbilical cord, amniotic fluid, placental tissue, and menstrual blood [20]. These multipotent stem cells have shown the capacity to restore ovarian function through homing, paracrine signaling, anti-inflammatory, anti-apoptotic and anti-fibrosis effects, in addition to promoting angiogenesis [20,110]. A systematic review of 41 animal and human clinical studies demonstrated that stem cell therapy may be useful in accelerating rejuvenation of ovarian tissues, regulating sex hormone levels and increasing fertility rate [111].

### 5.2. Intraovarian Platelet-Rich Plasma Injections

Intraovarian injection of PRP is currently explored as a novel regenerative treatment, particularly for women with diminished ovarian reserve or POI. Platelet-rich plasma is an autologous concentrate derived from fresh blood via centrifugation, leading to the production of plasma with a notably higher concentration of growth factors, hormones, and cytokines [112,113]. Those bioactive molecules contribute to angiogenesis, immunomodulation, cell proliferation, differentiation, and tissue regeneration [113,114,115]. When injected into the ovary, PRP may stimulate ovarian tissue regeneration and promote folliculogenesis, thereby restoring ovarian function [112]. Improvements of the hormonal profiles, including lower follicle-stimulating hormone and luteinizing hormone and higher anti-Müllerian hormone levels, in addition to increased antral follicle count, have been observed in human and animal models. In some cases, these changes were accompanied by spontaneous pregnancy or improved in vitro fertilization (IVF) results [112,113,114,115,116]. While initial observational studies and cohort data are promising, reliable clinical evidence for the efficacy of intraovarian PRP injections is still lacking, making this therapy not yet accepted in clinical practice [114].

### 5.3. Antioxidants

One of the most recognized contributors to ovarian aging is oxidative stress, largely driven by the accumulation of reactive oxygen species (ROS) [117,118,119]. Even though ROS are crucial for maintaining normal ovarian physiology, such as regulation of oocyte growth, meiosis, and ovulation, pathological states of oxidative stress in the body lead to accelerated apoptosis, telomere shortening, mitochondrial dysfunction, and inflammation [118,119]. These effects result in impaired folliculogenesis, reduced oocyte quality, and hastened ovarian senescence [117]. Thus, reducing oxidative stress in the ovaries appears to be critical for delaying ovarian aging, and antioxidants may be beneficial for maintaining reproductive longevity [119].

#### 5.3.1. Resveratrol

Resveratrol is a natural polyphenol with antioxidant, anti-inflammatory, antiaging, and antineoplastic properties [120]. It is found in red grapes, red wine, or peanuts [121,122]. Although the exact mechanism of anti-aging cellular processes remains unclear, in studies with animals, resveratrol has shown potential in delaying ovarian aging by scavenging ROS, regulating mitochondrial function, supporting oocyte maturation, reducing follicular atresia, and improving the ovarian microenvironment [120,121,122].

#### 5.3.2. Coenzyme Q10

Coenzyme Q10 (CoQ10) is a fat-soluble benzoquinone, found in almost all cell membranes and crucial for energy production and antioxidation [117,119,123]. Levels of CoQ10 gradually decline with age, especially in high-energy-demand tissues, including ovaries [123]. CoQ10 is studied in reproductive medicine for its ability to improve ovarian reserve and oocyte quality, resulting in better reproductive performance [117,124]. It has been confirmed that CoQ10 is present in human follicular fluid, and its level positively correlates with oocyte quality [124,125]. A 2024 meta-analysis of 20 studies showed that CoQ10 supplementation is associated with statistically significant improvement of the fertility of women with diminished ovarian reserve [117].

#### 5.3.3. Vitamins C and E

Vitamins C and E are potent natural antioxidants with an ability to scavenge free radicals, diminish DNA damage, inhibit lipid peroxidation, and protect cell membranes from injury [126]. Vitamin C, a water-soluble antioxidant, is able to regenerate oxidized vitamin E (α-tocopherol), while vitamin E, an oil-soluble antioxidant, primarily neutralizes lipid radicals during lipid peroxidation [22]. Animal studies have shown that oral vitamin C and E supplementation can mitigate aging-related decline in ovarian reserve and oocyte quality by reducing chromosomal aberrations and oocyte apoptosis [127].

#### 5.3.4. N-acetyl-L-cysteine

N-acetyl-L-cysteine (NAC) is a widely studied antioxidant and a precursor of reduced glutathione [128]. In reproductive biology, NAC can decrease telomere shortening, telomere fusion, and chromosomal instability in oocytes that are induced by oxidative stress [22]. Administration of low-dose NAC in mice not only enhanced telomerase activity but also improved oocyte quality in older mice and increased litter size [129]. A prospective study of advanced-age women undergoing IVF or intracytoplasmic sperm injection demonstrated that NAC improves ovarian responsiveness to gonadotropins and enhances the quality of blastocysts [128].

### 5.4. Metformin

Recently, metformin, a drug commonly prescribed for type 2 diabetes, has drawn interest for its potential antiaging properties, including its possible capability to delay ovarian aging [22]. Besides regulating glucose and insulin blood levels, metformin also downregulates insulin-like growth factor 1 (IGF-1) signaling, modulates metabolic pathways, reduces DNA damage, diminishes production of ROS, and even alters the microbiome [130]. These effects mirror those of caloric restriction, which has been shown to delay aging of the body, as well as to extend reproductive lifespan by slowing down the ovarian aging in animal models [22]. A study of long-term administration of metformin in mouse models showed an increase in primordial and primary follicles, maintained regular estrous cycles, and elevated estrogen levels. These effects were linked to increased expression of protein sirtuin 1 (SIRT1), a key regulator of cellular longevity, and reduced oxidative damage [130]. Another study with female mice discovered that metformin prevents age-related ovarian fibrosis by remodeling the extracellular matrix, controlling immune cell population, and promoting the clearance of senescence-associated fibroblasts [131]. While metformin has been extensively studied and used in females with polycystic ovary syndrome, in which it improves ovulatory function and hormonal balance [132,133], its effects on ovarian aging in healthy individuals remain under investigation [22].

### 5.5. Rapamycin

Rapamycin, an immunosuppressive drug and a selective inhibitor of mammalian target of rapamycin (mTOR), specifically the mTORC1, is a promising pharmacological ovarian-protective agent [134]. The mTOR pathway regulates follicular activation; therefore, increased mTOR activity in the oocyte prematurely stimulates primordial follicles, resulting in the depletion of the ovarian reserve [135]. By suppressing mTOR, rapamycin inhibits primordial follicle development and preserves them in quiescent state, thus helping to maintain follicular pool reserve [136]. Additionally, this mTOR inhibitor appears to be protective against chemotherapy-induced ovarian damage, indicating potential to preserve fertility in young female cancer patients receiving chemotherapy [134]. As animal studies demonstrate the ability of rapamycin to prolong ovarian lifespan [137,138], there is an ongoing randomized, placebo-controlled study called VIBRANT—Validating Benefits of Rapamycin for Reproductive Aging Treatment (NCT05836025)—to assess the potential of rapamycin in delaying ovarian aging in women.

### 5.6. Telomerase Activators

Telomeres are protective nucleoprotein structures at the ends of chromosomes that are essential for maintaining genome integrity [139]. Progressive telomere shortening with each cell division is one of the principal causes of cellular aging, eventually leading to senescence or apoptosis when critical length is reached [139,140]. There is a positive association between telomere length and ovarian reserve, as well as female reproductive longevity [138]. Telomere attrition can be counteracted by a ribonucleoprotein enzyme, telomerase, by adding telomeric repeats to the ends of chromosomes [140]. Telomerase is highly expressed in the ovaries, especially in granulosa cells, which undergo extensive proliferation and are exposed to hormones and other factors [141]. Shorter telomeres and decreased telomerase activity have been observed in conditions linked to female infertility, for example, POI [140]. Antioxidants NAC and resveratrol may delay ovarian aging not only by reducing ROS but also by increasing telomerase activity and lengthening telomeres [22]. However, concerns remain regarding the possibility of stimulating telomerase in dormant cancer cells, thereby increasing potential oncogenic risk [140].

### 5.7. Hormones

Hormones, such as melatonin and DHEA, have been studied for their potential to mitigate ovarian aging and improve reproductive outcomes in women with diminished ovarian reserve.

#### 5.7.1. Melatonin

Melatonin is a hormone secreted primarily by the pineal gland and is most known for regulating sleep and circadian rhythms. Melatonin is also synthesized by oocytes, granulosa cells and luteal cells, and its level in the human follicular fluid surpasses that in the blood [142]. By exhibiting strong antioxidant properties, melatonin reduces oxidative stress in the follicular environment and protects oocytes and granulosa cells from ROS [142,143,144]. Besides scavenging ROS, melatonin controls the activity of both pro-oxidant and antioxidant enzymes [142]. Other mechanisms by which melatonin delays ovarian aging include direct inhibition of follicle activation, growth, and atresia [143]; maintenance of telomere length; reduction in autophagy; and activation of cellular longevity pathways such as SIRT1 and SIRT3 [144]. A meta-analysis of seven randomized controlled trials with women undergoing IVF found that oral melatonin supplementation significantly increased the number of mature oocytes and showed a non-significant trend for higher clinical pregnancy rates [145].

#### 5.7.2. Dehydroepiandrosterone

Dehydroepiandrosterone is a weak androgen produced primarily by the adrenal gland and to a lesser extent by the ovarian theca cells. It is a precursor to sex steroids, such as testosterone and estradiol. Levels of circulating DHEA decline with age [146]. Dehydroepiandrosterone supplementation for women with diminished ovarian reserve may improve ovarian function and increase pregnancy rate by enhancing steroid hormone production, improving ovarian markers such as anti-Müllerian hormone and inhibin B, and increasing antral follicle count, potentially due to upregulation of IGF-1 [22,146,147]. In addition, DHEA can alter immune function, including CD4+/CD8+ T cell balance [148]. Studies with animal models support the potential anti-aging effects of DHEA on the ovary. In rats with diminished ovarian reserve, DHEA treatment improved hormone imbalances, reduced follicular atresia, raised ovarian volume and growing follicle number, and enhanced mitochondrial function [149]. Similarly, a study using perimenopausal rats showed improvement in the ovarian reserve parameters and pregnancy outcomes; however, the positive effects of DHEA appear to be dose dependent, as greater doses may induce polycystic-like ovarian morphology [150].

### 5.8. Stem Cell-Derived Extracellular Vesicles

Recent advances in nanotechnology have opened new possibilities for combating ovarian aging. Among these innovations, extracellular vesicles, particularly exosomes, have emerged as a promising natural nanocarrier system. Due to their biocompatibility with the immune system, low toxicity, ability to avoid phagocytosis, and capacity to cross biological barriers, exosomes serve as an effective vehicle for the targeted delivery of bioactive molecules [151,152]. Extracellular vesicles derived from mesenchymal stem cells of bone marrow, umbilical cord, adipose tissue, amniotic membrane, and menstrual blood have demonstrated potential to restore ovarian function in models of POI or chemotherapy-induced ovarian damage. By transferring functional microRNAs and proteins, extracellular vesicles suppress granulosa cell apoptosis, reduce oxidative stress, promote angiogenesis, improve folliculogenesis, and restore levels of follicle-stimulating hormone and estradiol [152,153]. However, further research is required before stem cell-derived extracellular vesicles can be translated into clinical practice.

### 5.9. Advanced Glycation Processes

Recent studies suggest that advanced glycation end products (AGE) may contribute to processes associated with ovarian aging, as they are found in increased amounts in aged ovaries. Both AGE and their receptors (RAGE) are expressed in granulosa, theca, and luteinized cells derived from the ovary. Accumulation of AGE within the ovarian follicular microenvironment may exacerbate age-related oxidative stress, impair glucose uptake by granulosa cells, and adversely affect follicular growth and ovulatory function. Although the clinical relevance of the AGE-RAGE axis in ovarian aging remains to be fully elucidated, this pathway represents a potential mechanism in reproductive aging and may offer new therapeutic targets [154,155,156].

## 6. Conclusions

As life expectancy continues to rise, women spend a significant part of their lives in the postmenopausal stage, making reproductive and menopausal health a cornerstone of lifelong well-being. Menopausal hormone therapy remains the gold standard for menopause symptom relief, yet contraindications, safety concerns, and individual preferences emphasize the need for effective alternatives. Complementary methods to ease symptoms of GSM include vaginal moisturizers and lubricants, hyaluronic acid in topical hydrating gels or intravaginal injections, local estrogen formulations, ospemifene, vaginal DHEA, oxytocin, laser therapy, and PRP vaginal injections. Vasomotor symptoms may be relieved by phytoestrogens and certain antidepressants.

Simultaneously, advances in fertility preservation techniques, including oocyte vitrification, ovarian tissue cryopreservation, in vitro follicle maturation, artificial ovary, artificial intelligence, and robotics, establish reproductive autonomy for women facing age-related ovarian reserve decline, POI, or gonadotoxic therapies.

Looking ahead, novel approaches, such as stem cell therapy, intraovarian PRP injections, antioxidants, metabolic modulators, telomerase activators, nanocarriers, and hormones other than female sex steroids, hold potential for delaying ovarian aging and prolonging reproductive longevity. While these innovations remain largely experimental, their continued exploration offers hope for a future where reproductive aging becomes a manageable, rather than inevitable, process.

## Figures and Tables

**Table 1:** Alternative Approaches to Menopausal Symptom Relief: Summary and Recommendations.

Category	Intervention	Main Indications	Safety Profile	Practical Recommendations
Local agents	Vaginal moisturizers and lubricants	Symptoms of GSM	Generally safe with minimal adverse effects	First-line therapies for GSM; moisturizers 2–3 x/week regardless of sexual activity; lubricants as needed during intercourse
	Hyaluronic acid	Symptoms of GSM	Generally well-tolerated and safe	Recommended for women preferring non-hormonal options; efficacy comparable to vaginal estrogen in mild–moderate GSM
	Local estrogen therapy	Symptoms of GSM	Generally safe; minimal systemic absorption at low doses	Gold standard for moderate–severe GSM when no other menopause symptoms are present
	Local DHEA therapy	Symptoms of GSM	Generally safe; serum hormone levels remain postmenopausal	Suitable for GSM with prominent sexual dysfunction; option when estrogen avoidance is preferred
	Vaginal oxytocin	Symptoms of GSM	Appears safe; limited long-term data	May be considered experimental/adjunctive; further studies needed before routine use
Pharmacological therapy	Ospemifene (SERM)	Symptoms of GSM	Generally well-tolerated and safe; possible vasomotor symptoms	Effective non-hormonal oral option for GSM; consider for women unsuitable for local estrogen
	SSRIs/SNRIs	Vasomotor symptoms	Generally well-tolerated; possible gastrointestinal symptoms, sleep changes	Recommended when hormone therapy is contraindicated; paroxetine and venlafaxine most studied
Devices and regenerative procedures	Laser therapy	Symptoms of GSM	Generally well-tolerated and safe; limited long-term data	Effective minimally invasive treatment for GSM; non-hormonal option for breast cancer survivors; fractional microablative CO_2_ and erbium:YAG lasers most studied
	PRP vaginal injections	Symptoms of GSM	Appears safe; limited long-term data	Recommended for women preferring non-hormonal options; further studies are needed
Non-pharmacological products	Phytoestrogens	Vasomotor symptoms	Generally safe	May be tried for mild symptoms; data on effectiveness inconclusive

DHEA—dehydroepiandrosterone; GSM—genitourinary syndrome of menopause; SERM—selective estrogen receptor modulator; SSRIs—selective serotonin reuptake inhibitors; SNRIs—serotonin/norepinephrine reuptake inhibitors; PRP—platelet-rich plasma.

**Table 2:** Established and Emerging Strategies for Extending Ovarian Function.

Intervention	Primary Target/Indication	Mechanism of Action	Clinical Application	Key Limitations
Oocyte vitrification	Age-related fertility decline; gonadotoxic therapy	Cryopreservation of mature oocytes using ultra-rapid cooling	Fertility preservation for personal or medical reasons	Age- and oocyte-number dependent outcomes; requires ovarian stimulation
Ovarian tissue cryopreservation	Prepubertal girls; urgent cancer treatment	Cryopreservation of ovarian cortex containing primordial follicles	Fertility and endocrine function restoration after autotransplantation	Surgical procedure; risk of malignant cell reintroduction
In vitro follicle maturation	Hematologic malignancies; high ovarian metastasis risk; avoidance of cancer cell reintroduction	Ex vivo maturation of primordial follicles	Experimental fertility preservation	Technically complex; limited human data
Artificial ovary	High ovarian metastasis risk; endocrine function restoration	Transplantation of isolated follicles within biocompatible scaffold	Experimental fertility and endocrine function restoration	Scaffold optimization; vascularization challenges
Artificial intelligence and robotics	Optimization of reproductive techniques	Data-driven decision support; precision surgery	Assisted reproduction and ovarian tissue procedures	Requires validation; access and cost constraints

**Table 3:** Experimental and Future Strategies to Delay Ovarian Aging.

Intervention	Main Biological Target/Indication	Proposed Anti-Aging Mechanism	Evidence Base
Stem cell therapy	Ovarian tissue regeneration	Paracrine signaling, anti-apoptotic and anti-inflammatory effects, promotion of angiogenesis	Animal and early human studies
Intraovarian PRP injection	Diminished ovarian reserve, POI	Bioactive factors-mediated folliculogenesis, angiogenesis, immunomodulation, cell proliferation, and tissue regeneration	Animal studies, observational and cohort human studies
Antioxidants (e.g., resveratrol, CoQ10, vitamins C and E, NAC)	Oxidative stress, mitochondrial dysfunction	ROS reduction, mitochondrial protection, anti-inflammatory and antineoplastic effects, improvement of follicular environment	Animal studies, preclinical and limited clinical studies
Metformin	Metabolic and oxidative pathways	Metabolic pathways and IGF-1 signaling regulation, ROS reduction, SIRT1 activation, ovarian fibrosis prevention	Animal studies, data of women with PCOS
Rapamycin	mTOR signaling	Preservation of primordial follicle quiescence	Animal studies, ongoing RCT
Telomerase activators	Telomere attrition	Telomere length maintenance	Animal studies
Melatonin	Oxidative stress, circadian regulation	Antioxidant action, follicle protection, telomere length maintenance, autophagy reduction, SIRT1 and SIRT3 activation	Animal and clinical studies
DHEA	Diminished ovarian reserve	Steroidogenesis enhancement, IGF-1 signaling upregulation, immune function alteration	Animal and clinical studies
Stem cell-derived extracellular vesicles	Granulosa cell survival, angiogenesis	miRNA and protein delivery	Preclinical studies

CoQ10—coenzyme Q10, DHEA—dehydroepiandrosterone, IGF-1—insulin-like growth factor 1, mTOR—mammalian target of rapamycin, NAC—N-acetyl-l-cysteine, PCOS—polycystic ovary syndrome, POI—primary ovarian insufficiency, PRP—platelet-rich plasma, RCT—randomized controlled trial, ROS—reactive oxygen species, SIRT—sirtuin.

## Data Availability

No new data were created or analyzed in this study.

## Abbreviations

AGE	Advanced glycation end products
CoQ10	Coenzyme Q10
DHEA	Dehydroepiandrosterone
GSM	Genitourinary syndrome of menopause
HRT	Hormone replacement therapy
IGF-1	Insulin-like growth factor 1
IVF	In vitro fertilization
mTOR	Mammalian target of rapamycin
NAC	N-acetyl-l-cysteine
POI	Primary ovarian insufficiency
PRP	Platelet-rich plasma
ROS	Reactive oxygen species
SIRT	Sirtuin
